# Aspirin attenuates YAP and β-catenin expression by promoting β-TrCP to overcome docetaxel and vinorelbine resistance in triple-negative breast cancer

**DOI:** 10.1038/s41419-020-2719-2

**Published:** 2020-07-13

**Authors:** Ji Ma, Zhenhai Fan, Qiulin Tang, Hongwei Xia, Tao Zhang, Feng Bi

**Affiliations:** 1 Department of Medical Oncology and Laboratory of Molecular Targeted Therapy in Oncology, State Key Laboratory of Biotherapy, West China Hospital, Sichuan University, No. 37, Guoxue Lane, 610041 Chengdu, Sichuan China; 2 Key Laboratory of Cell Engineering of Guizhou, The Affiliated Hospital of Zunyi Medical College, No. 149, Dalian Road, 573003 Zunyi, Guizhou China; 3 Department of Oncology, The General Hospital of Western Theater Command, No. 270, Tianhui Road, 610041 Chengdu, Sichuan Province PR China

**Keywords:** Oncogenes, Breast cancer

## Abstract

The use of aspirin has been associated with reduced breast cancer risk, but it is litter known if aspirin overcomes chemoresistance in triple-negative breast cancer (TNBC). Herein, we demonstrated that changes in the expression of Yes-associated protein (YAP) and β-catenin might be a promising predictive biomarker for neoadjuvant chemotherapy sensitivity in TNBC patients. Inhibition of YAP or β-catenin enhanced the cytotoxicity of the anti-microtubule agents docetaxel and vinorelbine against drug-resistant TNBC cells as well as the sensitivity of these cells to the agents in vitro and in vivo. Interestingly, aspirin not only significantly inhibited the growth of TNBC cells, but also attenuated YAP and β-catenin expression by upregulating the E3 ubiquitin ligase β-TrCP to abolished docetaxel and vinorelbine resistance. The combination of aspirin and docetaxel or vinorelbine remarkably inhibited the growth of drug-resistant TNBC cells in vitro and in vivo. Moreover, TNBC patients with high YAP and/or β-catenin expression had a higher risk of relapse or mortality than patients with low YAP and/or β-catenin expression. Collectively, our study discovered a novel role of aspirin based on its anticancer effect, and put forward some possible mechanisms of chemoresistance in TNBC. The combined use of aspirin and anti-microtubule drugs presented several promising therapeutic approaches for TNBC treatment.

## Introduction

Breast cancer had one of the highest incidences and one of the highest mortality rates of cancers that affect females in 2018^1^. Despite many advances in targeted molecular therapy, the mortality rate of breast cancer remains very high. Triple-negative breast cancer (TNBC) accounts for 15–20% of all breast cancer cases and is a subtype of breast cancer characterized by the lack of oestrogen receptor (ER), progesterone receptor (PR) and ErbB-2/human epidermal growth factor receptor 2 (HER-2) expression^2^. Due to the lack of effective therapeutic targets, TNBC is associated with more aggressive behaviours, earlier relapse and poorer prognosis. Chemotherapy remains the mainstream treatment for TNBC patients^2^. Docetaxel- and vinorelbine-based regimens have proven to be greatly effective in early and advanced TNBC, respectively^3^. However, docetaxel or vinorelbine resistance is very common. Therefore, more effective therapeutic strategies for TNBC are urgently needed.

Many epidemiological studies have found that tumour mortality decreases exponentially with an increase in aspirin intake in several cancers, including colon, breast, lung, and prostate cancers and cancers of other organs^4–6^. The cancer-preventing effects of aspirin and other non-steroidal anti-inflammatory drugs (NSAIDs) are well established for breast cancer, and NSAIDs could impede the development of ER-positive breast cancer through the inhibition of aromatase^7^. The latest study found that regular aspirin use was associated with a 39% reduced risk of breast cancer, and the strength of these associations did not differ by familial risk or mutation status^8^. Intriguingly, in *BRCA1* or *BRCA2* germline mutation carriers, aspirin use was associated with a 27% or 20% reduced risk of breast cancer, respectively^8^. Moreover, TNBC is a subtype of breast cancer with the highest *BRCA* mutation rate^9^. The retrospective analysis found that aspirin use improved disease-free survival and reduced the risk of distant metastases in stage II and stage III TNBC patients^10^, but it is unclear whether aspirin could increase the sensitivity to chemotherapy drugs or reverse drug resistance in TNBC.

The Hippo pathway is an evolutionarily conserved signalling pathway that plays important roles in organ size control, tissue regeneration, stem cell self-renewal and tumorigenesis^11^. YAP is the major downstream effector of Hippo signalling, and members of the Hippo pathway can act as transcriptional coactivators to promote the expression of their target genes involved in proliferation and survival^12^. Accumulating evidence suggests oncogenic roles of YAP in cancer progression, but the role of YAP remains controversial in different subtypes of breast cancer^13^. The Wnt/β-catenin pathway plays a major role in embryonic growth and leads to tumorigenesis when aberrantly activated^14^. Many studies have indicated that this pathway is abnormally activated in the progression of several cancers, including breast cancer^15^. β-Catenin is stabilized and translocated to the nucleus, where it acts as a cofactor to activate the expression of target genes implicated in cell growth^16^. In addition, it has been reported that the Hippo/YAP and Wnt/β-catenin pathways are mutually regulated and that their crosstalk plays a vital role in many physiological and pathological processes^17,18^. However, whether YAP or β-catenin is involved in chemotherapy resistance in breast cancer remains elusive.

In this study, we confirm that YAP and β-catenin mediates docetaxel and vinorelbine resistance in TNBC and that aspirin could enhance the cytotoxicity of anti-microtubule drugs and reverse drug resistance. The specific mechanism is that aspirin impairs YAP and β-catenin by upregulating the E3 ubiquitin ligase β-TrCP to overcome docetaxel and vinorelbine resistance. Moreover, TNBC patients with high YAP and/or β-catenin expression had a higher risk of relapse or mortality than patients with low YAP and/or β-catenin expression. The combination of aspirin and anti-microtubule drugs might be a promising therapeutic strategy for TNBC.

## Materials and methods

### Tissue samples and the study cohort

Thirty pairs of paraffin-embedded breast carcinoma and adjacent normal breast tissue samples, as well as 112 paraffin-embedded breast carcinoma samples, were collected and these tissues were made into tissue microarray slides (Shanghai Outdo Biotech Co., Ltd). These samples were prospectively obtained from patients with breast cancer who underwent resection from January 2005 to December 2011 and were followed up for 2.1–11 years. The tissues larger than 5 cm from the tumor margin were selected and obtained as the adjacent normal tissues and these tissues were diagnosed and confirmed by pathologists as the normal tissues. Fresh tumor tissues were obtained from 40 patients with breast cancer undergoing neoadjuvant chemotherapy. All these patients received four cycles of AC (doxorubicin 60 mg/m^2^ and cyclophosphamide 600 mg/m^2^) followed by four cycles of docetaxel (100 mg/m^2^). This study was approved by the Ethics Committee of the 940th Hospital of Joint Logistics Support Force of Chinese People’s Liberation Army.

### Immunohistochemistry

Immunohistochemistry (IHC) was performed on all tissue samples using biotin-streptavidin HRP detection systems. After deparaffinization with xylene and dehydration in a graded alcohol series, the tissue sections were subjected to antigen retrieval by microwaving in sodium citrate buffer for 10 min and then inhibiting endogenous peroxidase activity. After nonspecific binding was blocked, the slides were incubated with YAP (1:200) and β-catenin (1:200) antibody (Santa Cruze, USA), Ki67 (1:300, MXB biotechnologies, China) in phosphate-buffered saline (PBS) overnight at 4 °C in a humidified container. Biotinylated secondary antibodies (Zhongshan Golden Bridge Biotechnology, China) were then used according to the manufacturer’s recommendations. The sections were incubated with HRP-streptavidin conjugates appropriate for detecting these proteins. The brown color indicative of peroxidase activity was developed by incubation with 0.1% 3,3′-diaminobenzidine (Zhongshan Golden Bridge Biotechnology) in PBS with 0.05% H_2_O_2_ for 5 min at room temperature. The appropriate positive and negative controls were included in each run of IHC.

### Staining evaluation

An immunoreactivity score system based on the proportion and intensity of positively stained cells was applied. The two extensional standards taken were as follows: (1) number of positively stained cells: ≤5%, 0; 6–25%, 1; 26–50%, 2; 51–75%, 3; and >75%, 4; and (2) staining intensity: colorless, 0; pallideflavens, 1; yellow, 2; and brown, 3. Scores (1) and (2) were multiplied, and the staining grade was classified as absent (score 0), weak (score 1–4), moderate (score 5–8) or strong (score 9–12). Specimens were rescored independently by two pathologists. Moderate or strong tumor immunostaining was classified as having high expression, and absent or weak tumor immunostaining was classified as having low expression.

### RT-PCR

The total RNA from each sample was extracted using NucleoZOL reagent (MACHEREY-NAGEL, Germany) in accordance with the recommended protocol. Retrotranscription was performed with the Reverse Transcriptase M-MLV (Takara, Japan). The RT-PCR reactions were performed with a SYBR Premix Ex Taq™ kit (Takara, Japan) on a Bio-Rad CFX96 Touch system (Bio-Rad, USA). The primers used were as follows: YAP, forward: GGTGCCACTGTTAAGGAAAGG, reverse: GTGAGGCCACAGGAGTTAGC; β-catenin, forward: CATTACAACTCTCCACAACC3, reverse: CAGATAGCACCTTCAGCAC; β-actin, forward: TGGCACCCAGCACAATGAA, reverse: CTAAGTCATAGTCCGCCTAGAAGCA. The data were analyzed using the 2^−ΔΔCt^ method.

### Cell cultures and reagents

The human TNBC cell lines MDA-MB-231 and MDA-MB-468 were from ATCC and stored in our laboratory. The docetaxel or vinorelbine-resistant MDA-MB-231 cell lines (MDA-MB-231/DR or MDA-MB-231/VR) were treated and established with docetaxel or vinorelbine for about 6 months in our laboratory. All these cell lines were cultured in DMEM medium supplemented with 10% fetal bovine serum (HyClone, USA) in 5% CO_2_ at 37 °C. Docetaxel and vinorelbine were purchased from Aosaikang and Haosen in China, respectively. Aspirin was purchased from Sigma in USA. The following antibodies were used for IHC and western blotting: YAP (Abcam, UK), β-catenin (Santa Cruze, USA), β-TrCP, Bcl-l2, Bax (CST, USA), C-Myc, CyclinD1 (Epitomics, USA), Ki67 (MXB biotechnologies, China), GAPDH (Biostar, China).

### Cell survival, colony formation and apoptosis assays

For cell survival assay, the cells were seeded in 96-well plates for 24 h and were allowed to adhere overnight in regular growth media. After treatment with the indicated drugs or transfection for 48 h, the relative cell proliferation was measured using the Cell Counting Kit-8 (Dojingdo, Kumamoto, Japan). For colony formation assay, a total of cells (1 × 10^3^) were seeded in a 35 mm dishes. After 10 days, the resulting colonies were rinsed with PBS, fixed with methanol at −4 °C for 5 min, and stained with Giemsa (Sigma) for 20 min. For apoptosis assay, we used the Annexin V-FITC Apoptosis detection kit (Dojindo Molecular Technologies, Japan). Cells were collected and washed twice with PBS and then resuspended in 500 μl of staining solution containing fluorescein isothiocyanate (FITC)-conjugated annexin V antibody (5 μl). Subsequent to the staining, the cells were incubated with 10 μl of propidium iodide (PI) for 5 min on ice in the dark. The analyses were performed using a Navios flow cytometer (Beckman Coulter).

### DNA replication activity and migration assays

For DNA replication activity assay, we used BrdU (5-bromo-2-deoxyuridine) kit (Ribobio, China). Cells grown on coverslips were treated with different drugs for 48 h and then incubated with BrdU for 1 h and stained with an anti-BrdU antibody according to the manufacturer’s instructions. The results were analyzed using a fluorescence microscope (Olympus). For accessing cell migration ability assay, we used scratch wounding migration assay. The cells were cultured in six-well plates and reached 90% confluence; a scratch wound was created using a pipette tip. To avoid the effects of cell proliferation, the cells were treated by 5 µM mitomycin C for 2 h. The wound edges were photographed with a Nikon Eclipse TE 2000-U (Nikon, Japan), and the scratch widths were analyzed using ImageJ software.

### Western blot

The cells were lysed in RIPA buffer with protease inhibitors and phosphatase inhibitors (Calbiochem, Darmstadt, Germany). The protein concentration was determined by the Bradford protein assay kit (BioRad). Proteins were resolved by SDS-PAGE and transferred to polyvinyl difluoride (PVDF) membranes (Millipore). The blots were probed with the different primary antibodies and species-matched secondary antibodies. The expression of the proteins was detected using the BioRad semidry transfer system.

### Xenograft studies in nude mouse

A total of 5 × 10^6^ cells (in 100 μl PBS) were injected into the abdominal mammary fat pad of 4-week-old female nude mice (Dashuo, China). After the tumour volumes exceeded ~100 mm^3^, mice were randomized into nine groups (*n* = 5 per group): Group 1 (control) mice received intratumoural negative control shRNA lentiviral vector (1 × 10^8^ TU, 50 μl) injections and intraperitoneal phosphate-buffered saline (50 μl) injections. Group 2 (sh-YAP) mice received intratumoural sh-YAP lentiviral vector (1 × 10^8^ TU, 50 μl) injections. Group 3 (sh-β-catenin) mice received intratumoural sh-β-catenin lentiviral vector (1 × 10^8^ TU, 50 μl) injections. Group 4 (Doc) mice received intraperitoneal docetaxel injections at a dose of 8 mg/kg twice per week. Group 5 (Vin) mice received intraperitoneal vinorelbine injections at a dose of 2 mg/kg twice per week. Group 6 (sh-YAP + Doc) mice received sh-YAP lentiviral vector and docetaxel injections. Group 7 (sh-β-catenin + Doc) mice received sh-β-catenin lentiviral vector and docetaxel injections. Group 8 (sh-YAP + Vin) mice received sh-YAP lentiviral vector and vinorelbine injections. Group 9 (sh-β-catenin + Vin) mice received sh-β-catenin lentiviral vector and vinorelbine injections. Three weeks after treatment, mice were sacrificed, and tumours were resected. Additionally, a nude mouse study of drug-resistant TNBC cells was also carried out following the above methods. After the tumour volumes exceeded ~100 mm^3^, mice were randomized into groups (*n* = 5 per group): Group 1 (control) mice received intragastric and intraperitoneal phosphate-buffered saline injections. Group 2 (Doc) mice received intraperitoneal docetaxel injections at a dose of 8 mg/kg twice per week. Group 3 (Vin) mice received intraperitoneal vinorelbine injections at a dose of 2 mg/kg twice per week. Group 4 (Doc + Asp) mice received intraperitoneal docetaxel injections and intragastric aspirin at 50 mg/kg/d. Group 5 (Vin + Asp) mice received intraperitoneal vinorelbine injections and intragastric aspirin at 50 mg/kg/d. Tumour growth was monitored by caliper measurements using the following formula: *V* = (length × width^2^)/2, where *L* and *W* represent the length and width, respectively, and the survival of mice was analysed by Kaplan–Meier analysis. The animal experiments were approved by the institutional review board of West China Hospital of Sichuan University. All efforts were made to minimize the animals’ suffering and reduce the number of animals used.

### Statistical analysis

Statistical analyses were performed with GraphPad Prism version 5.0 (La Jolla, CA, USA) and SPSS statistical software, version 22.0 (SPSS Inc, Chicago, IL). Data from in vitro experiments were analysed statistically using a one-way analysis of variance (ANOVA), an independent samples *t-*test or Student’s *t*-test. All data are expressed as the mean ± standard deviation. In the clinical specimen study, the associations between YAP or β-catenin expression and categorical variables were analysed using Pearson’s chi-square test as appropriate. Correlations between YAP or β-catenin expression and the expression of other molecules were analysed by using the Spearman correlation test. Kaplan–Meier analysis was used to evaluate disease-free survival and overall survival. The effects of different variables on outcomes were assessed using a univariate Cox regression model to select proper variables for multivariate analysis and build a more reliable model. A multivariate Cox proportional hazards model was used to identify factors affecting DFS and OS. *P* values of less than 0.05 were considered statistically significant.

## Results

### Expression of YAP and β-catenin in breast cancer and adjacent normal breast tissues

**Fig. 1:**
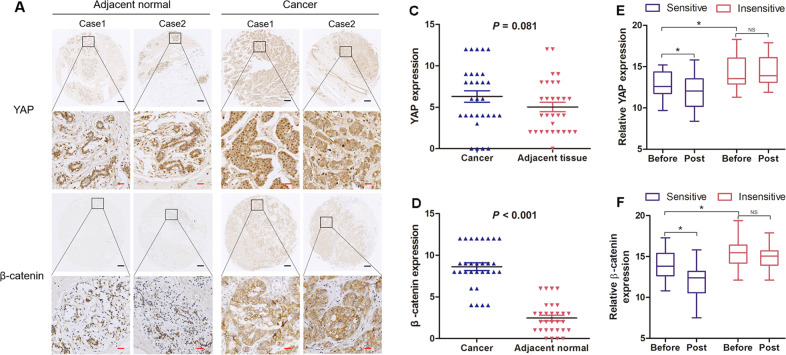
Different expression levels of YAP and β-catenin in human breast cancer. **a**, **b** Immunostained tissue microarray slides for YAP and β-catenin in breast cancer and normal tissues were analysed. Black scale bar: 200 µM and red scale bar: 25 µM. **c**, **d** Relative expression levels of YAP and β-catenin in human breast cancer and adjacent normal tissue are shown. The immunoreactivity score distributions of cancer and adjacent normal tissue are represented with blue and red closed arrows, respectively. The horizontal lines represent the means; error bars represent the SD from 30 samples. **e**, **f** Dynamic expression changes in YAP or β-catenin before and after NCT in TNBC. RT-PCR was employed to detect the RNA expression levels of YAP and β-catenin before and after NCT. The RNA expression levels of chemotherapy-sensitive and chemotherapy-insensitive breast cancer tissues are represented with blue and red boxes, respectively. Relative YAP expression: in the sensitive group Before vs Post (*P* = 0.009); Before in sensitive group vs Before in insensitive group (*P* = 0.017). Relative β-catenin expression: in the sensitive group Before vs Post (*P* = 0.003); Before in sensitive group vs Before in insensitive group (*P* = 0.007). Before, before chemotherapy. Post, after chemotherapy. Statistical significance was determined using Student’s *t* test analysis.

Oncogenic roles of β-catenin in breast cancer has been confirmed, but the role of YAP remains controversial^13^. To explore the roles of YAP and β-catenin in the carcinogenesis of human breast cancer, we investigated the expression of YAP and β-catenin by IHC in 30 pairs of breast carcinoma and adjacent normal breast tissue specimens. Positive staining of YAP was found predominantly in the cytoplasm and nucleus of both normal breast cells and breast cancer cells (Fig. 1a). Conversely, strong positive staining of β-catenin was observed in the cytoplasm and cytosolic membrane of tumour cells, and weaker staining was found in noncancerous breast cells (Fig. 1b). Indeed, in normal tissues and cancer tissues, the means of the immunoreactivity score of YAP IHC staining were 5.03 and 6.30 (Fig. 1c), respectively, and the means of the IHC staining score of β-catenin were 2.47 and 8.63 (Fig. 1d), respectively. There was no significant difference in YAP expression between the cancer specimens and the adjacent normal tissues (*P* = 0.081), while β-catenin expression was higher in cancer tissues than in the paired adjacent normal tissues (*P* < 0.001).

### Changes in the expression of YAP and β-catenin in TNBC treated with neoadjuvant chemotherapy

It remains elusive that whether YAP or β-catenin is involved in chemotherapy resistance in breast cancer, especially in TNBC. Therefore, we examined the expression of YAP and β-catenin in TNBC before and after neoadjuvant chemotherapy (NCT). As shown in Fig. 1e, before chemotherapy, YAP expression was significantly upregulated in the insensitive group (*P* = 0.017); in the sensitive group, YAP expression was decreased after chemotherapy (*P* = 0.009), but in the insensitive group, there was no significant difference between the values before and after chemotherapy. The changes in expression of β-catenin in TNBC treated with NCT were similar to the changes seen in YAP expression (Fig. 1f). Before chemotherapy, β-catenin expression was significantly upregulated in the insensitive group (*P* = 0.007); in the sensitive group, β-catenin expression was decreased after chemotherapy (*P* = 0.003). The data implied that changes in the expression of YAP or β-catenin might be a promising predictive biomarker for response in TNBC patients with NCT. More importantly, YAP or β-catenin might also be involved in chemotherapy resistance in TNBC treatment.

### YAP and β-catenin mediated the growth inhibition of docetaxel and vinorelbine in TNBC cells

**Fig. 2:**
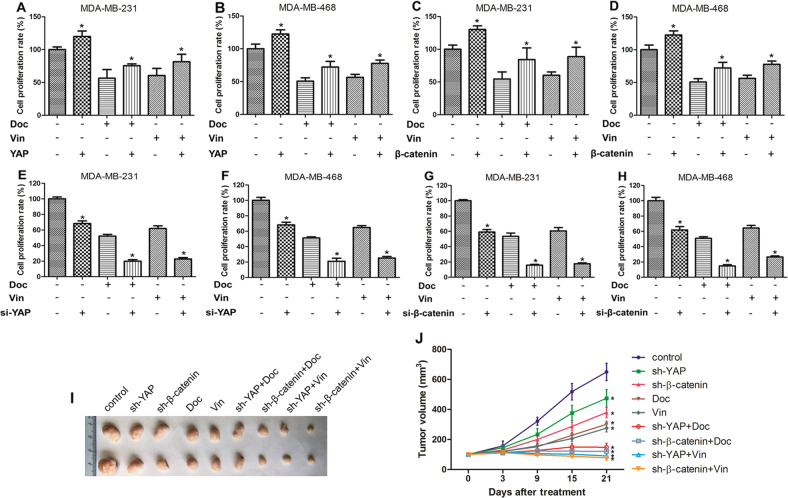
YAP and β-catenin affect the growth-inhibiting effects of docetaxel and vinorelbine on TNBC cells. **a** CCK-8 assay for MDA-MB-231 and MDA-MB-468 cells cultured with control, pCMV-Flag-YAP-5SA (activated YAP), 5 nM docetaxel (Doc), 5 μM vinorelbine (Vin), pCMV-Flag-YAP-5SA and Doc or Vin for 48 hours. Control group *vs* YAP group (*P* = 0.005); Doc group vs Doc + YAP group (*P* = 0.030); Vin group *vs* Vin + YAP group (*P* = 0.036). The rest of the CCK-8 assays were similar to those described above. **b** Control group vs YAP group (*P* = 0.004); Doc group vs Doc + YAP group (*P* = 0.005); Vin group vs Vin + YAP group (*P* < 0.001). **c** pcDNA3.0-β-catenin was used for β-catenin overexpression. Control group *vs* β-catenin group (*P* < 0.001); Doc group vs Doc + β-catenin group (*P* = 0.029); Vin group vs Vin + β-catenin group (*P* = 0.010). **d** Control group *vs* β-catenin group (*P* = 0.004); Doc group vs Doc + β-catenin group (*P* = 0.005); Vin group vs Vin + β-catenin group (*P* < 0.001). **e**, **f** siRNA-YAP was used for YAP knockdown. Control group vs si-YAP group (*P* < 0.001); Doc group vs Doc + si-YAP group (*P* < 0.001); Vin group vs Vin + si-YAP group (*P* < 0.001). **g**, **h** Control group vs si-β-catenin group (*P* < 0.001); Doc group vs Doc + si-β-catenin group (*P* < 0.001); Vin group vs Vin + si-β-catenin group (*P* < 0.001). The data are presented as the mean ± SD of experiments performed in triplicate. Tumour formation assays in nude mice subcutaneously injected with MDA-MB-231 cells. The specific treatment was performed as described in the “Materials and Methods”. **i** Photograph of the tumour tissue. **j** Tumour growth was assessed every 3 or 6 days until treatment day 21 by measuring two perpendicular diameters and calculating the volume. Doc, docetaxel; Vin, vinorelbine; sh-YAP, lentiviral vector of YAP shRNA; sh-β-catenin, lentiviral vector of β-catenin shRNA. The data presented are means ± SDs; error bars represent the SD from five mice. Control group vs different treatment groups (*P* < 0.001). Statistical significance was determined using Student’s *t* test and one-way ANOVA test analysis.

Docetaxel and vinorelbine are the main drugs for breast cancer treatment, and both drugs exert their anticancer effects by inhibiting the formation of microtubules in tumour cells. To investigate whether YAP or β-catenin affect the inhibitory effects of docetaxel or vinorelbine in TNBC cells, CCK-8 assays were performed. As shown in Fig. 2a–d, docetaxel or vinorelbine alone could inhibit the proliferation of TNBC MDA-MB-231 and MDA-MB-468 cells, but after combining the two drugs with overexpression of YAP or β-catenin, the survival rates of the two cell lines were increased. After treatment with chemotherapy and inhibition of YAP or β-catenin expression, the survival of these cells was suppressed significantly (Fig. 2e–h). The in vivo experiments were grouped as follows: a control group, an intratumoural YAP shRNA (sh-YAP) lentiviral vector injection group, an intratumoural β-catenin shRNA (sh-β-catenin) lentiviral vector injection group, a chemotherapy groups, and lentiviral vector injection combined with chemotherapy groups. We employed these treatments on preestablished human MDA-MB-231 breast tumours (~100 mm^3^) grown in nude mice. As shown in Fig. 2i–j, tumour growth was inhibited in both the lentiviral vector injection and chemotherapy groups and remarkably in the lentiviral vector injection combined with chemotherapy groups. Taken together, these data suggested that YAP or β-catenin inhibition enhanced the cytotoxicity of docetaxel and vinorelbine against TNBC cells in vitro and in vivo.

### Upregulation of YAP or β-catenin with docetaxel or vinorelbine resistance in TNBC cells

**Fig. 3:**
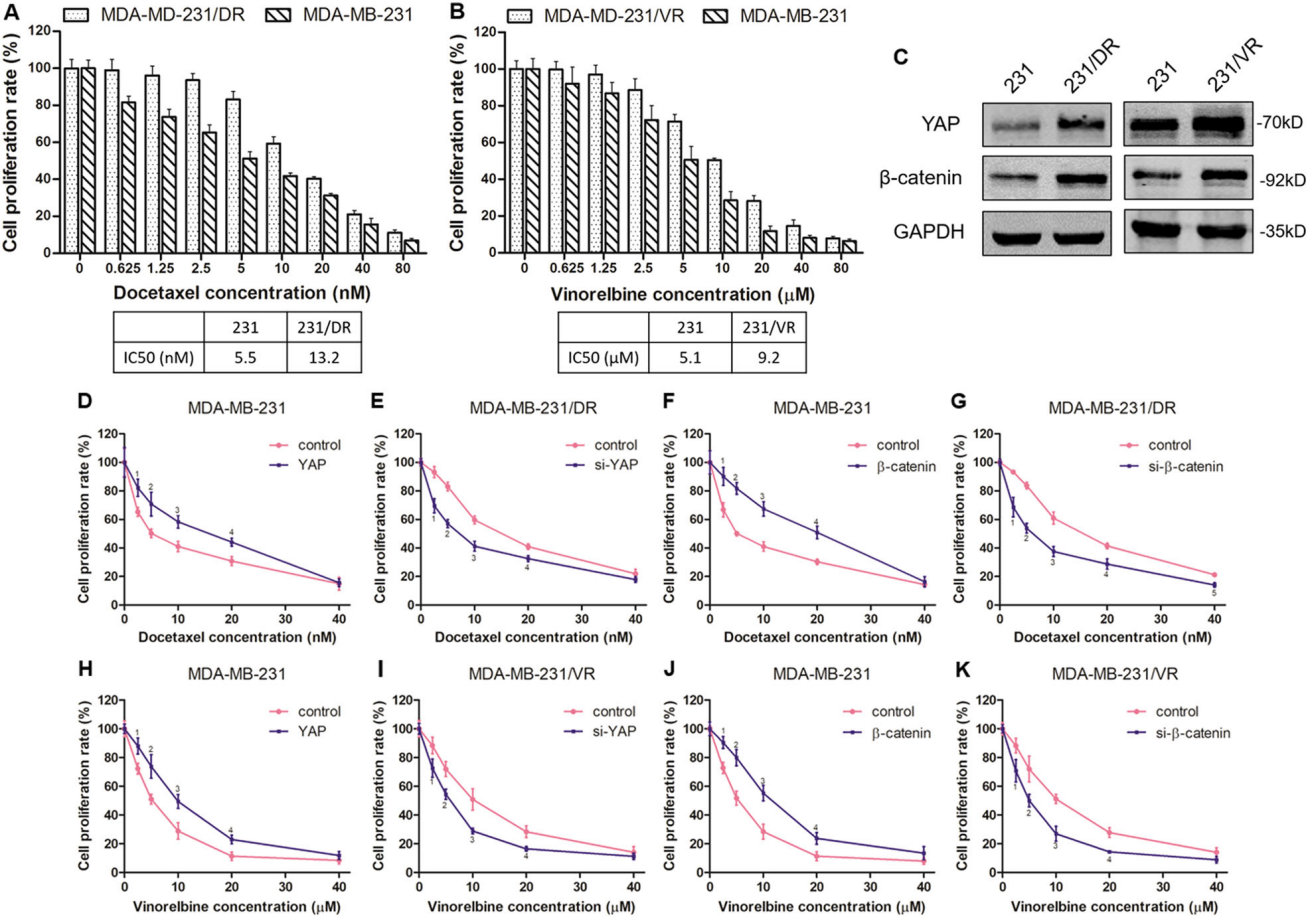
YAP and β-catenin promote docetaxel and vinorelbine resistance in TNBC cells. **a**, **b** The CCK-8 assay showed that MDA-MB-231/DR and MDA-MB-231/VR cells were more resistant to docetaxel and vinorelbine, respectively, than MDA-MB-231 cells. **c** The protein expression of YAP and β-catenin in drug-resistant and drug-sensitive cells was detected by western blot assays. **d** CCK-8 assays for MDA-MB-231 cells transfected with control or pCMV-Flag-YAP-5SA plasmid cultured with docetaxel at different concentrations for 48 h. Control group vs YAP group (^1^*P* = 0.013, ^2^*P* = 0.016, ^3^*P* = 0.006, ^4^*P* = 0.005). The rest of the CCK-8 assays were similar to those described above. **e** siRNA-YAP was used for YAP knockdown. Control group vs si-YAP group (^1^*P* = 0.003, ^2^*P* < 0.001, ^3^*P* = 0.002, ^4^*P* = 0.008). **f** pcDNA3.0-β-catenin was used for β-catenin overexpression. Control group vs β-catenin group (^1^*P* = 0.008, ^2^*P* < 0.001, ^3^*P* = 0.002, ^4^*P* = 0.002). **g** siRNA-β-catenin was used for β-catenin knockdown. Control group *vs* si-β-catenin group (^1^*P* = 0.004, ^2^*P* < 0.001, ^3^*P* = 0.002, ^4^*P* = 0.006, ^5^*P* = 0.002). **h** pCMV-Flag-YAP-5SA was used for activated YAP. Control group *vs* YAP group (^1^*P* = 0.016, ^2^*P* = 0.012, ^3^*P* = 0.009, ^4^*P* = 0.009). **i** siRNA-YA*P* was used for YAP knockdown. Control group vs si-YAP group (^1^*P* = 0.034, ^2^*P* = 0.009, ^3^*P* = 0.008, ^4^*P* = 0.009). **j** pcDNA3.0-β-catenin was used for β-catenin overexpression. Control group *vs* β-catenin group (^1^*P* = 0.006, ^2^*P* = 0.003, ^3^*P* = 0.003, ^4^*P* = 0.014). **k** siRNA-β-catenin was used for β-catenin knockdown. Control group vs si-β-catenin group (^1^*P* = 0.031, ^2^*P* = 0.019, ^3^*P* = 0.002, ^4^*P* = 0.003). The data are presented as the mean ± SD of experiments performed in triplicate. Statistical significance was determined using Student’s *t* test analysis.

To assess whether YAP or β-catenin mediated docetaxel or vinorelbine resistance in TNBC cells, we designed the following assays. First, we established an MDA-MB-231 docetaxel-resistant cell line (MDA-MB-231/DR) and a vinorelbine-resistant cell line (MDA-MB-231/VR). As shown in Fig. 3a, b, the IC50 value of docetaxel in MDA-MB-231/DR cells was higher than that in MDA-MB-231 cells (13.2 nM vs 5.5 nM). Similarly, the IC50 value of vinorelbine in MDA-MB-231/VR cells was also higher than that in MDA-MB-231 cells (9.2 µM vs 5.1 µM). Next, we found that both YAP and β-catenin were upregulated in drug-resistant cells compared with non-resistant cells (Fig. 3c and S1). These data suggested that YAP or β-catenin might be correlated with docetaxel or vinorelbine resistance in TNBC cells. Therefore, we further explored the effect of YAP or β-catenin expression on docetaxel or vinorelbine sensitivity in TNBC cells. As shown in Fig. 3d–g, overexpression of YAP or β-catenin significantly promoted MDA-MB-231 cell resistance to docetaxel treatment. In contrast, knockdown of YAP or β-catenin increased docetaxel sensitivity in MDA-MB-231/DR cells. The experiment in the vinorelbine group showed similar results to that in the docetaxel group (Fig. 3h–k). Our data showed that YAP or β-catenin overexpression could significantly increase the resistance of TNBC cells to docetaxel or vinorelbine, while YAP or β-catenin repression increased the sensitivity of the drug-resistant cells to docetaxel or vinorelbine.

### Aspirin reversed docetaxel and vinorelbine resistance in TNBC cells through downregulation of YAP and β-catenin

**Fig. 4:**
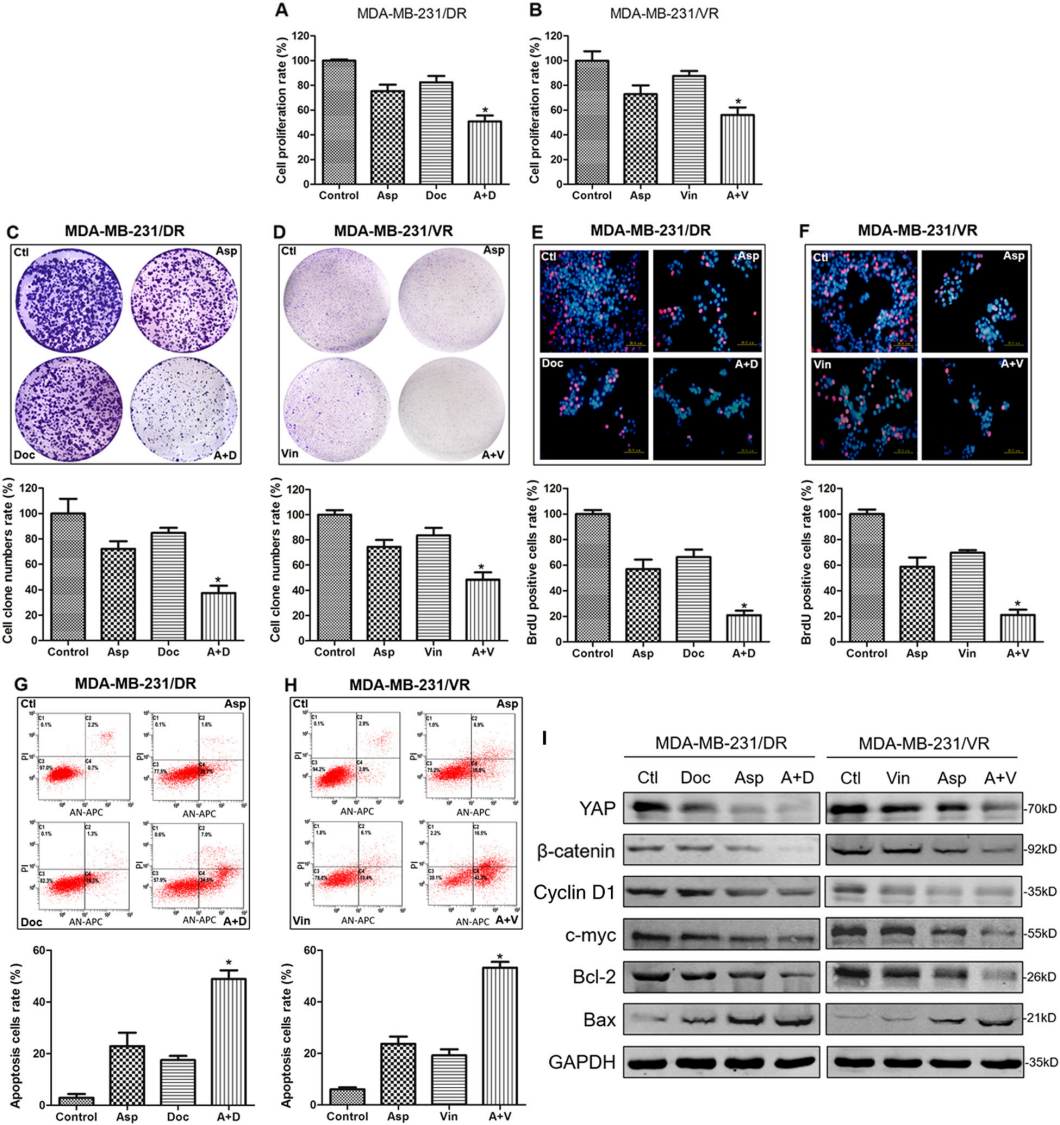
Aspirin suppressed docetaxel and vinorelbine resistance in TNBC cells. CCK-8, colony formation, DNA replication activity and apoptosis assays for MDA-MB-231/DR and MDA-MB-231/VR cells cultured with control, 5 mM aspirin (Asp), 5 nM docetaxel (Doc), 5 μM vinorelbine (Vin), aspirin plus docetaxel (A + D), and aspirin plus vinorelbine (A + V). The rest of the assays were performed as and the groups were similar to those described above. **a** CCK-8 assay for MDA-MB-231/DR. Control vs A + D (*P* < 0.001); Asp vs A + D (*P* = 0.004); Doc+ A + D (*P* = 0.002). **b** CCK-8 assay for MDA-MB-231/VR. Control vs A + D (*P* = 0.001); Asp vs A + D (*P* = 0.036); Doc+ A + D (*P* = 0.002). **b** CCK-8 assay for MDA-MB-231/VR. Control vs A + D (*P* < 0.001); Asp *vs* A + D (*P* = 0.004); Doc+ A + D (*P* = 0.002). **c** Colony formation assay for MDA-MB-231/DR. Control vs A + D (*P* = 0.001); Asp vs A + D (*P* = 0.002); Doc+ A + D (*P* < 0.001). **d** Colony formation assay for MDA-MB-231/VR. Control vs A + D (*P* < 0.001); Asp vs A + D (*P* = 0.005); Doc+ A + D (*P* = 0.002). **e** DNA replication activity assay for MDA-MB-231/DR. DNA replication activity is a critical index for cell growth. BrdU, a synthetic thymidine analogue that binds to replicating DNA, was used to examine the rate of DNA replication. A red color indicates cells in the DNA replication phase, and a blue color indicates the cell nucleus. Scale bar: 20 µM. Control *vs* A + D (*P* < 0.001); Asp vs A + D (*P* = 0.002); Doc+ A + D (*P* < 0.001). **f** DNA replication activity assay for MDA-MB-231/VR. Control vs A + D (*P* < 0.001); Asp vs A + D (*P* = 0.001); Doc+ A + D (*P* < 0.001). **g** Apoptosis assay for MDA-MB-231/DR. Control vs A + D (*P* < 0.001); Asp vs A + D (*P* = 0.002); Doc+ A + D (*P* < 0.001). **h** Apoptosis assay for MDA-MB-231/VR. Control vs A + D (*P* < 0.001); Asp vs A + D (*P* < 0.001); Doc+ A + D (*P* < 0.001). **i** Western blot for YAP, β-catenin, cyclin D1, c-myc, Bcl-2 and Bax in MDA-MB-231/DR and MDA-MB-231/VR cells cultured with control, 5 mM aspirin (Asp), 5 nM docetaxel (Doc), 5 μM vinorelbine (Vin), aspirin plus docetaxel (A + D), or aspirin plus vinorelbine (A + V) for 48 h. The data are presented as the mean ± SD of experiments performed in triplicate. Statistical significance was determined using Student’s *t* test analysis.

To assess the anticancer effect of aspirin on TNBC cells, the proliferation, colony formation and migration assays were employed. The data indicated that aspirin can inhibit the growth of TNBC cells remarkably (Fig. S2). Next, we analysed whether aspirin could reverse drug resistance in TNBC cells. As shown in Fig. 4a, b, docetaxel or vinorelbine alone had little effect on the survival of MDA-MB-231/DR or MDA-MB-231/VR cells, but the combination of aspirin with the two agents significantly inhibited cell survival compared with the docetaxel or vinorelbine single treatments. Similar effects were also found in colony formation and BrdU assays (Fig. 4c–f). The apoptosis assay demonstrated that single treatment with docetaxel or vinorelbine produced a low rate of apoptosis, but the combination with aspirin induced apoptosis in almost 50% of the drug-resistant cells (Fig. 4g, h). To further investigate the mechanisms of the combined inhibition, we then analysed cell extracts for the expression of proliferation- and apoptosis-related markers. Western blot results showed that YAP and β-catenin expression was only slightly suppressed by the three drugs alone, whereas the combination drug treatment resulted in significant inhibition. Similar inhibitory effects of these agents were found on the expression of cyclin D1 and c-myc. Finally, the combination of aspirin with chemotherapy led to significant induction of the pro-apoptotic gene bax and inhibition of the anti-apoptotic gene bcl-2 (Fig. 4I). Together, these data indicated that aspirin could reverse docetaxel and vinorelbine resistance in TNBC cells, and this process might be mediated by YAP and β-catenin.

### Aspirin sensitizes TNBC to docetaxel and vinorelbine treatment in vivo

**Fig. 5:**
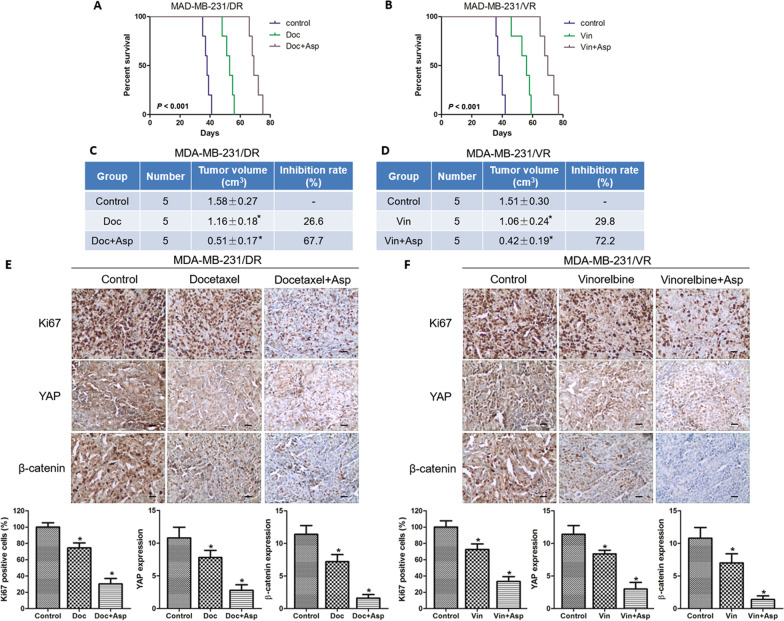
Antitumour effect of the combination of aspirin and docetaxel or vinorelbine in a drug-resistant nude mouse model. Tumour formation assays in nude mice subcutaneously injected with MDA-MB-231/DR or MDA-MB-231/VR cells. When the tumours reached ~100 mm^3^, the mice were divided into a control group and groups receiving docetaxel (Doc, 8 mg/kg), aspirin (Asp, 50 mg/kg), and/or vinorelbine (Vin, 2 mg/kg). **a**, **b** The survival of mice was analysed by Kaplan–Meier analysis. **c**, **d** The tumour sizes were measured, and the inhibition rates were calculated. MDA-MB-231/DR group (Control vs Doc, *P* = 0.020, Control vs Doc + Asp, *P* < 0.001). MDA-MB-231/VR group (Control vs Vin, *P* = 0.030, Control vs Vin + Asp, *P* < 0.001). **e**, **f** Representative immunohistochemical staining results for Ki67, YAP and β-catenin in xenograft tumour tissues. The graph shows the immunoreactivity scores of Ki67, YAP and β-catenin in each group. Scale bar: 25 µM. In MDA-MB-231/DR group, the *P* values were shown, as fellow: Ki67 (Control *vs* Doc, *P* < 0.001; Control vs Doc + Asp, *P* < 0.001), YAP (Control vs Doc, *P* = 0.009; Control *vs* Doc+Asp, *P* < 0.001), β-catenin (Control vs Doc, *P* < 0.001; Control *vs* Doc+Asp, *P* < 0.001). In MDA-MB-231/VR group, the *P* values were shown, as fellow: Ki67 (Control *vs* Doc, *P* < 0.001; Control vs Doc + Asp, *P* < 0.001), YAP (Control vs Doc, *P* = 0.002; Control vs Doc+Asp, *P* < 0.001), β-catenin (Control vs Doc, *P* = 0.004; Control vs Doc+Asp, *P* < 0.001). The data are presented as the mean ± SD of experiments performed. Statistical significance was determined using Student’s *t* test and log-rank analysis.

The above data indicated that aspirin could sensitize drug-resistant TNBC cells to docetaxel or vinorelbine in vitro, but whether these combined strategies also function in vivo was unknown. We then established drug-resistant MDA-MB-231 breast tumours grown in nude mice and divided the mice into three groups: the control group, the chemotherapy group, and the aspirin combined with chemotherapy group. As shown in Fig. 5a–d, the chemotherapy group that was treated with docetaxel or vinorelbine only achieved arrest of tumour growth (26.6% or 29.8% decrease, respectively). The combined group that was treated with docetaxel plus aspirin or vinorelbine plus aspirin achieved a significant arrest of tumour growth (67.7% or 72.2% decrease, respectively). Furthermore, the control group had a shorter survival time than the chemotherapy group, whereas the aspirin combined with chemotherapy group had a longer survival time than the chemotherapy group. Finally, we also analysed the protein levels of Ki67, YAP and β-catenin by IHC. In Fig. 5e, f, the positive staining of Ki67, YAP or β-catenin was weaker in the chemotherapy group than in the control group. However, the protein expression levels were weaker in the single chemotherapy group than those in the aspirin combined with chemotherapy group. These data suggested that aspirin could increase the sensitivity of drug-resistant tumours to chemotherapy drugs through YAP and β-catenin inhibition in vivo.

### Aspirin decreased YAP or β-catenin expression by promoting β-TrCP expression

**Fig. 6:**
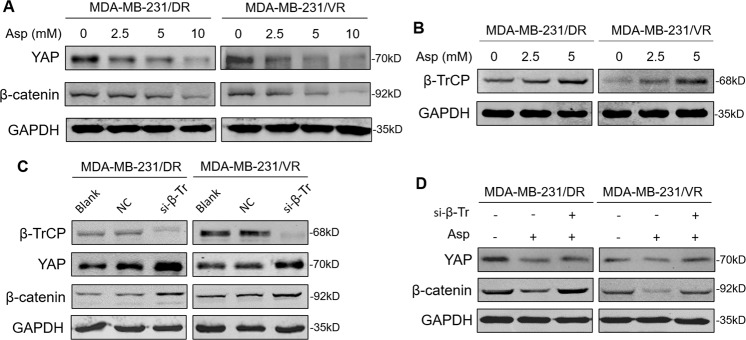
Aspirin inhibited YAP and β-catenin by upregulating β-TrCP. **a** The cells were cultured with different concentrations of aspirin (0, 2.5, 5 and 10 mM) for 48 h. Then, a western blot assay was employed to detect the protein expression of YAP and β-catenin. **b** β-TrCP protein was detected by western blot after treatment with different concentrations of aspirin (0, 2.5 and 5 mM). **c** The cells were divided into different groups: blank group (cultured with normal medium), NC (negative control) group (transfected with negative control siRNA), and si-β-TrCP group (transfected with siRNA for β-TrCP). The protein expression levels of β-TrCP, YAP and β-catenin were examined by western blot assay in the above cells. **d** The cells were divided into different groups: control group (cultured with DMSO and transfected with negative control siRNA), aspirin group (cultured with 5 mM aspirin), and aspirin + si-β-TrCP group (cultured with 5 mM aspirin and transfected with siRNA for β-TrCP). The protein expression levels of YAP and β-catenin were examined by western blot assay in the above cells and performed in in triplicate.

β-TrCP is an E3 ubiquitin ligase that regulates the expression of a variety of proteins^19^. YAP is phosphorylated by Hippo/LATS signaling kinase, causing YAP to remain in the cytoplasm^20^. β-TrCP catalyses YAP ubiquitination, ultimately leading to YAP degradation^20^. β-TrCP also acts as a ubiquitin ligase that directly recognizes and degrades phosphorylated β-catenin^21^. In this part of study, the specific mechanism by which aspirin inhibits YAP or β-catenin was explored. First, we found that aspirin could decrease YAP or β-catenin expression, and as the concentration of aspirin increased, the inhibitory effects became more significant (Fig. 6a). Moreover, aspirin increased the expression of β-TrCP (Fig. 6b). Further study showed that a small interfering RNA for β-TrCP (si-β-TrCP) inhibited the expression of β-TrCP and augmented the expression of YAP and β-catenin (Fig. 6c). Finally, aspirin impaired YAP or β-catenin expression, but this inhibitory effect of aspirin was weakened after knockdown of β-TrCP by si-β-TrCP (Fig. 6d). These data revealed that aspirin decreased YAP and β-catenin expression by promoting β-TrCP expression and this may be a possible mechanism of aspirin for overcoming the docetaxel- and vinorelbine-resistance in TNBC (Fig. S3).

### Association between YAP or β-catenin expression and the prognosis of breast cancer patients

**Fig. 7:**
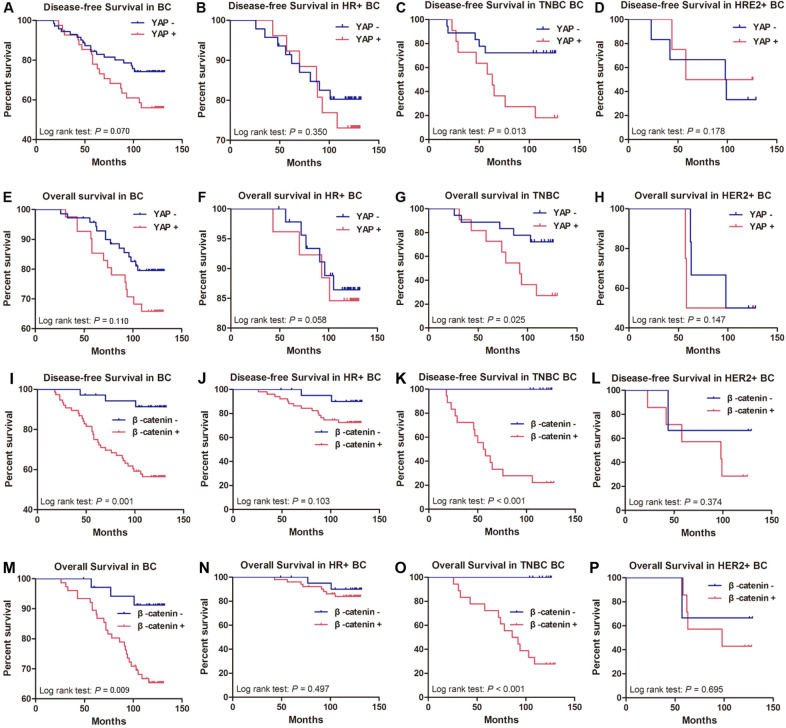
Association between YAP or β-catenin expression and survival in breast cancer patients. Kaplan–Meier analysis of disease-free survival (DFS) and overall survival (OS) was carried out according to the YAP or β-catenin expression levels in breast cancer subtypes. DFS: **a**, **i** Total breast cancer (BC). **b**, **j** Hormone receptor-positive breast cancer (HR+). **c**, **k** Triple-negative breast cancer (TNBC). **d**, **l** HER2-positive breast cancer (HER2+). OS: **e**, **m** Total breast cancer (BC). **f**, **n** Hormone receptor-positive breast cancer (HR+). **g**, **o** Triple-negative breast cancer (TNBC). **h**, **p** HER2-positive breast cancer (HER2+). Statistical significance was determined using log-rank analysis.

First, we analysed the histopathological characteristics of 112 breast cancer samples with available YAP or β-catenin protein status. The correlations between YAP or β-catenin expression and different clinical histopathological factors are presented in Table 1. Significant correlations were found between high YAP expression and smaller tumour size (*P* = 0.018) and positive lymph node metastasis status (*P* = 0.036). Moreover, high β-catenin expression was correlated with positive lymph node metastasis status (*P* = 0.028), high histological grade (*P* = 0.035), and advanced TNM staging (*P* = 0.040). The correlation coefficients are shown in Table S1. Next, we used Kaplan–Meier curves and a log-rank test to analyse disease-free survival (DFS) and overall survival (OS) rates among patients with breast cancer after stratification by YAP or β-catenin expression and breast cancer molecular subtype. The results showed that patients with low YAP expression in breast cancer tissues had no better DFS or OS than those with high YAP expression (Fig. 7a, e). Moreover, breast cancer patients with high β-catenin expression had poorer DFS and OS than patients with low β-catenin expression (Fig. 7i, m). Similarly, YAP and β-catenin expressions were not correlated with DFS or OS in HR+ patients (Fig. 7BFJN) or HER2+ patients (Fig. 7DHLP), but both of them were significantly correlated with DFS and OS in TNBC patients (Fig. 7CGKO). This means that TNBC patients with high YAP or β-catenin expression had a higher risk of relapse or mortality than those with low YAP or β-catenin expression. Among the patients with positive coexpression of YAP and β-catenin, the worst OS and DFS values were observed. In addition, low β-catenin levels were associated with long OS and DFS in both patients with high and patients with low YAP levels (Fig. S4). These results indicated that the combination of high expression of YAP and β-catenin defined a subgroup of breast cancer patients with extremely poor outcomes. At last, we next investigated the prognostic value of clinicopathological parameters using a univariate Cox regression model (Table S2). The significant parameters were selected for multivariate analysis (Table S3). In a multivariate analysis, ER was a significant independent predictor of favourable DFS and OS among all patients, whereas old age at diagnosis and high β-catenin expression were independent predictors of poor DFS and OS in this population. However, YAP expression, tumour size, lymph node metastasis, histological grade, TNM stage, and PR and HER2 status had no prognostic value for DFS and OS in the overall group.

**Table 1:** Association of YAP or β-catenin expression with clinical pathologic characteristics in breast cancer specimens.

Variable	No.	YAP expression			β-catenin expression		
		Low	High	*P* value ^a^	Low	High	*P* value ^a^
*Age(years)*							
≤50	43	25	18	0.362	14	29	0.971
>50	69	46	23		22	47	
*Tumor size*							
≤2 cm	44	22	22	0.018	14	30	0.953
>2 cm	68	49	19		22	46	
*Lymph node metastasis*							
Negative	61	44	17	0.036	25	36	0.028
Positive	51	27	24		11	40	
*Histological grade*							
I–II	75	49	26	0.544	29	46	0.035
III	37	22	15		7	30	
*TNM stage*							
I–II	72	50	22	0.074	28	44	0.040
III–IV	40	21	19		8	32	
*ER status*							
Negative	42	26	16	0.800	15	27	0.531
Positive	70	45	25		21	49	
*PR status*							
Negative	61	41	20	0.359	18	43	0.514
Positive	51	30	21		18	33	
*HER2 status*							
Negative	91	57	34	0.730	28	63	0.517
Positive	21	14	7		8	13	
*Molecular subtype*							
HR+	73	47	26	0.950	22	51	0.740
HER2+	10	6	4		3	7	
TNBC	29	18	11		11	18	

^a^The Pearson’s chi-square test was used for statistical analyses. *P* values < 0.05 were considered statistically significant.

## Discussion

The Hippo pathway is an evolutionarily conserved signalling pathway that restricts organ size and is found in organisms ranging from Drosophila to mammals^11^. The core upstream components of the Hippo pathway comprise several tumour suppressors, including Mst1/2, Sav1/WW45, Lats1/2, and Mob1^22^. These proteins act in a kinase cascade that culminates in the phosphorylation and inactivation of YAP/TAZ^22^. In most types of tumours, YAP acts as an oncogene to promote the expression of its target genes involved in cell proliferation and survival^23^. Our laboratory has previously identified a novel positive feedback loop involving YAP and CD44, in which CD44 functions as both an upstream regulator and a downstream effector of YAP through RhoA regulation in hepatocellular carcinoma (HCC)^24^. We also found that the EGFR-PI3K-PDK1 pathway could activate YAP signalling, and inhibition of YAP enhanced the cytotoxicity of EGFR pathway inhibitors^25^. Moreover, we also observed that MEK nuclear localization, which is an alternative mechanism of KRAS-targeted-drug resistance, promotes YAP stability by sequestering β-TrCP in KRAS mutant cancer cells^26^. However, the roles and regulatory mechanisms of YAP remain largely unknown in some tumours, such as breast cancer.

Whether YAP functions as a tumor suppressor or an oncogene remains controversial in breast cancer. Some studies suggested that YAP expression was decreased in breast cancer and associated with favorable prognosis, and based on these data it has been suggested that YAP may act as a tumor suppressor in breast cancer^27–30^. In contrast, another study suggested that YAP may act as an oncoprotein in breast cancer and this conclusion was drawn based on the observation that YAP expression was increased in certain breast cancers and promoted the tumor growth and radiation resistance of breast cancer^31,32^. In order to clarify these inconsistencies, we initially examined the expression of YAP in normal breast and breast cancer tissues and found that YAP expression in breast cancer tissues was higher than that in normal tissues, but there was no significant difference. Then, our data suggested that high YAP expression was correlated with smaller tumor size and positive lymph node metastasis and had not worse DFS or OS than those with low YAP expression in all breast cancer patients. Notably, subgroup analysis found that high YAP expression was correlated significantly with poor DFS or OS in TNBC patients, while there was no difference in HR+ and HER2+ subtypes. Therefore, YAP may mainly act as a tumor-promoting factor in TNBC, and could act as a promisingly prognostic marker for TNBC.

Wnt/β-catenin signalling has been demonstrated to contribute to tumorigenesis and cancer stem cell plasticity^16^. β-Catenin is stabilized and translocated to the nucleus, where it acts as a cofactor to activate the expression of target genes implicated in cell cycle progression, survival, angiogenesis, and metabolism^33^. The interactions between the Hippo/YAP and Wnt/β-catenin pathways play a crucial role in many physical and pathological processes^34^. Intriguingly, the crosstalk between the two pathways is context-dependent and can result in synergism. YAP has been shown to activate the Wnt/β-catenin cascade by promoting the nuclear translocation of the tyrosine phosphatase SHP2^35^. β-Catenin activation could promote YAP stabilization by inhibiting its proteolysis^36^. Moreover, β-catenin and YAP interact physically and are activated in most human hepatoblastoma tissues, and overexpression of activated forms of the two proteins in livers leads to rapid liver tumour development in mice^37^. Our study showed that β-catenin was overexpressed in breast cancer tissues compared with paired adjacent normal tissues and acted as an independent predictor of poor DFS and OS in breast cancer, especially for TNBC. Our results suggest that the combination of high expression of YAP and β-catenin defines a subgroup of breast cancer patients with extremely poor outcomes.

Docetaxel- and vinorelbine-based regimens have been confirmed to be greatly effective in breast cancer patients^38^. However, drug resistance remains the key problem for treatment with docetaxel and vinorelbine. Our clinical data suggested that YAP/β-catenin might be involved in chemotherapy resistance in TNBC treatment. In the cellular experiments, we found that YAP and β-catenin counteracted the inhibitory effects of docetaxel and vinorelbine on TNBC cells. Interestingly, in a nude mouse model, tumour growth was remarkably inhibited after combined treatment with knockdown of YAP or β-catenin and chemotherapy drugs. We investigated whether YAP or β-catenin mediated docetaxel and vinorelbine resistance in TNBC cells and found that both YAP and β-catenin were key modulators of docetaxel and vinorelbine resistance. In MDA-MB-231 docetaxel- and vinorelbine-resistant cell lines, repression of YAP or β-catenin increased the sensitivity of the drug-resistant cells to docetaxel and vinorelbine. Until now, there has been little study on YAP and docetaxel resistance, and the existing studies showed that YAP expression was elevated in docetaxel-resistant prostate cancer cells and that knockdown of YAP suppressed the migration and invasion of docetaxel-resistant prostate cancer cells^39^. Our data were consistent with these findings, implying that YAP may be a novel regulator of docetaxel-resistance. To our knowledge, this is the first report about YAP and β-catenin participating in vinorelbine resistance and YAP or β-catenin inhibition enhancing the sensitivity of vinorelbine-resistant breast cancer cells.

In recent years, many clinical epidemiological studies have found that with an increase in aspirin intake, tumour mortality decreases exponentially in patients with colon, breast, lung, and prostate cancers and cancers of other organs^5,40^. Other studies found that regular aspirin use was associated with a reduced risk in breast cancer patients, especially for high-risk populations with familial risk or a mutated status^8^. In addition, aspirin could also overcome tamoxifen resistance in oestrogen receptor-positive breast cancer cells^41^. Aspirin also induced autophagy to affect the proliferation, apoptosis and drug-resistance of tumour cells^42,43^. It is unclear whether aspirin could overcome docetaxel or vinorelbine resistance. Here, we reported for the first time that aspirin reversed docetaxel and vinorelbine resistance in vitro and in vivo in TNBC, and one possible mechanism was that aspirin reduced the expression of YAP and β-catenin by promoting β-TrCP, an E3 ubiquitin ligase. At present, it is controversy about which doses of aspirin could achieve the best antitumor effect. Different studies used different dose settings^4^. The aspirin concentrations (1–5 mM) used were consistent with measurements of aspirin in the serum of patients treated for chronic inflammatory diseases^44,45^. Hence, it is a very complicated process that aspirin exerts the antitumor effect in the human body and the cellular or animal studies do not fully reflect what happens in the human body. On the one hand, it is related to metabolism and distribution of aspirin in the body. On the other hand, different cancers show the different responses to aspirin. The laboratory studies just provide the possible mechanisms and directions, but it is still necessary to future study in the clinic and laboratory.

β-TrCP regulates the expression of a variety of proteins^19^. YAP is phosphorylated by Hippo/LATS signaling kinase, causing YAP to remain in the cytoplasm^20^. β-TrCP catalyses YAP ubiquitination, ultimately leading to YAP degradation^20^. β-TrCP also acts as a ubiquitin ligase that directly recognizes and degrades phosphorylated β-catenin^21^. Our previous study also showed that inhibition of β-TrCP promotes YAP stability and carcinogenicity^26^. Interestingly, in this study we found that aspirin can upregulate β-TrCP expression to inhibit YAP and β-catenin expression and it is a possible antitumour mechanism of aspirin. Some studies found that dephosphorylation by PP2A destroys the β-TrCP binding site created by phosphorylation. As a result, β-catenin is prevented from been recruited to the β-TrCP complex for ubiquitin conjugation, leading to its stabilization and high levels of accumulation^46^. Interestingly, in another study, aspirin acted through PP2A inhibition to increase β-catenin phosphorylation thereby reducing Wnt/β-catenin pathway activity, and aspirin acted on the Wnt/β-catenin pathway via a proteasome-dependent mechanism induced by β-TrCP^47^. Our results seem to be consistent with the inhibitive effects of aspirin on β-catenin through β-TrCP. In brief, we think that on the one hand, aspirin can inhibit PP2A to make β-TrCP bind to the phosphorylation of β-catenin and promote the degradation of β-catenin; on the other hand, aspirin may upregulate β-TrCP expression directly or indirectly to promote the degradation of β-catenin and this specific mechanism need further study.

In conclusion, this work demonstrated that both YAP and β-catenin affect the efficacy and mediate the resistance of docetaxel and vinorelbine in TNBC cells in vitro and in vivo. To our knowledge, this is the first report showing that aspirin can reverse docetaxel and vinorelbine resistance in TNBC cells in vitro and in vivo. It also suggests a possible mechanism by which aspirin impairs the expression of YAP and β-catenin by promoting β-TrCP to overcome drug resistance. Therefore, the combined use of aspirin and docetaxel or vinorelbine could be promising clinical approaches for TNBC treatment.

## Supplementary information

FigureS1

FigureS2

FigureS3

FigureS4

FigureS5

Table.S1

Table.S2

Table.S3

Supplementary figure legends
