# Supplementary figures and images for: Aspirin attenuates YAP and β-catenin expression by promoting β-TrCP to overcome docetaxel and vinorelbine resistance in triple-negative breast cancer
Source: Cell Death Dis. 2020 Jul 13;11(7):530. doi: 10.1038/s41419-020-2719-2 (PMC7359325; doi:10.1038/s41419-020-2719-2)

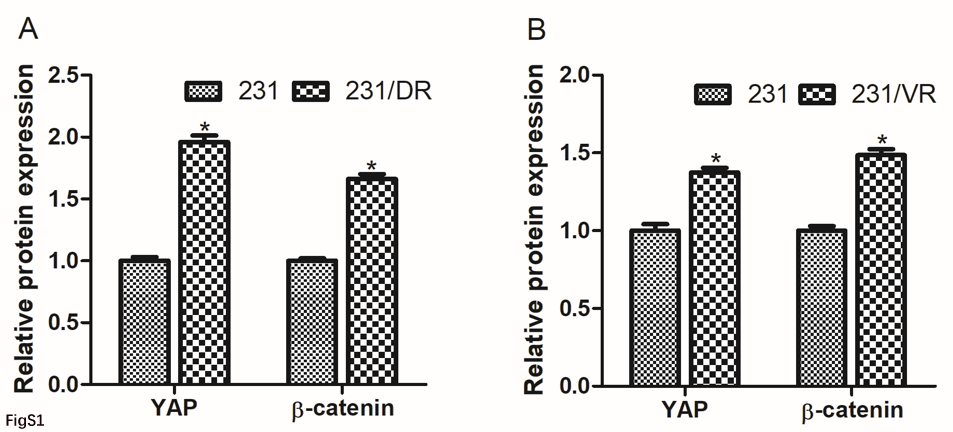

Supplement: Supplementary file 1 — FigureS1 [file 41419_2020_2719_MOESM1_ESM.tif]

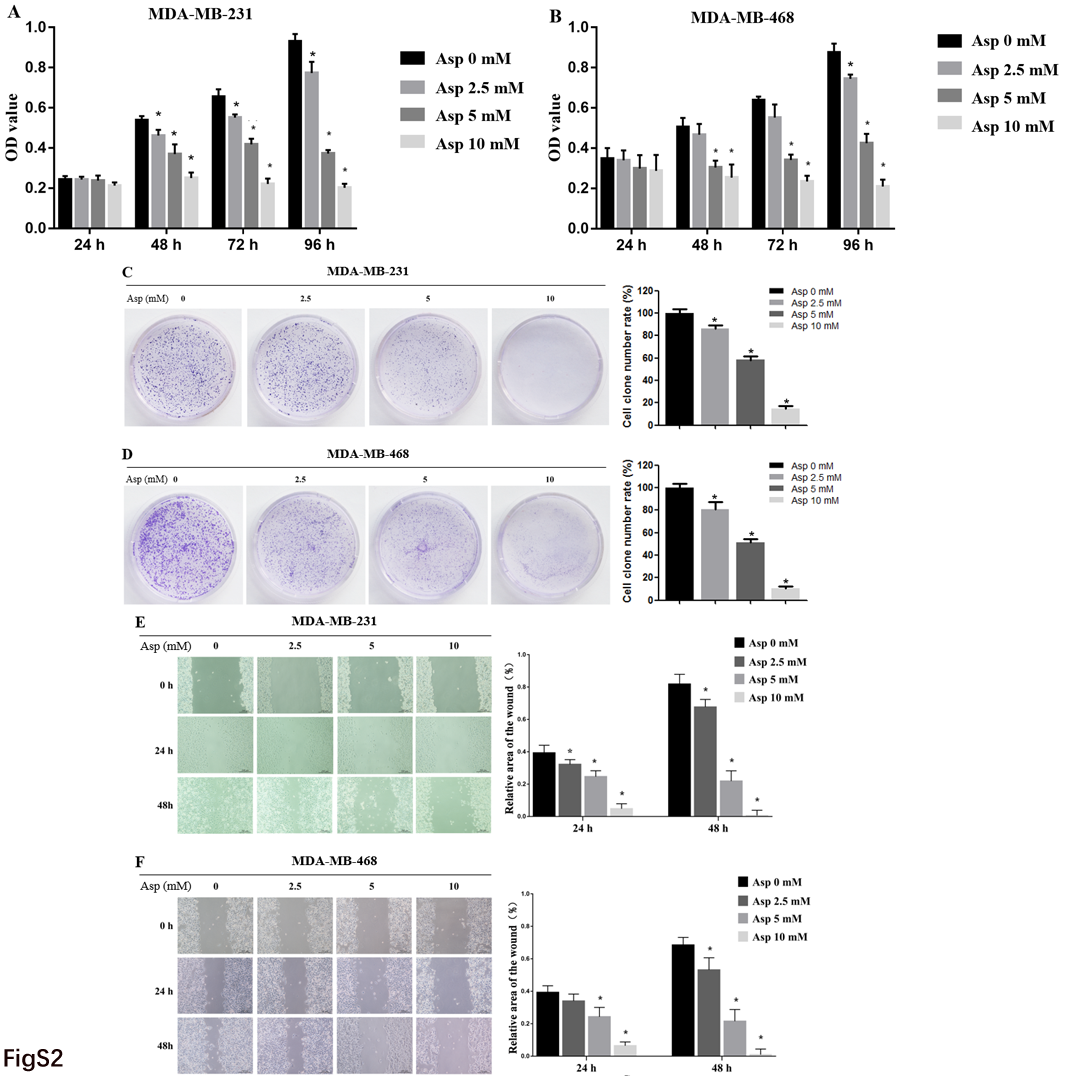

Supplement: Supplementary file 2 — FigureS2 [file 41419_2020_2719_MOESM2_ESM.tif]

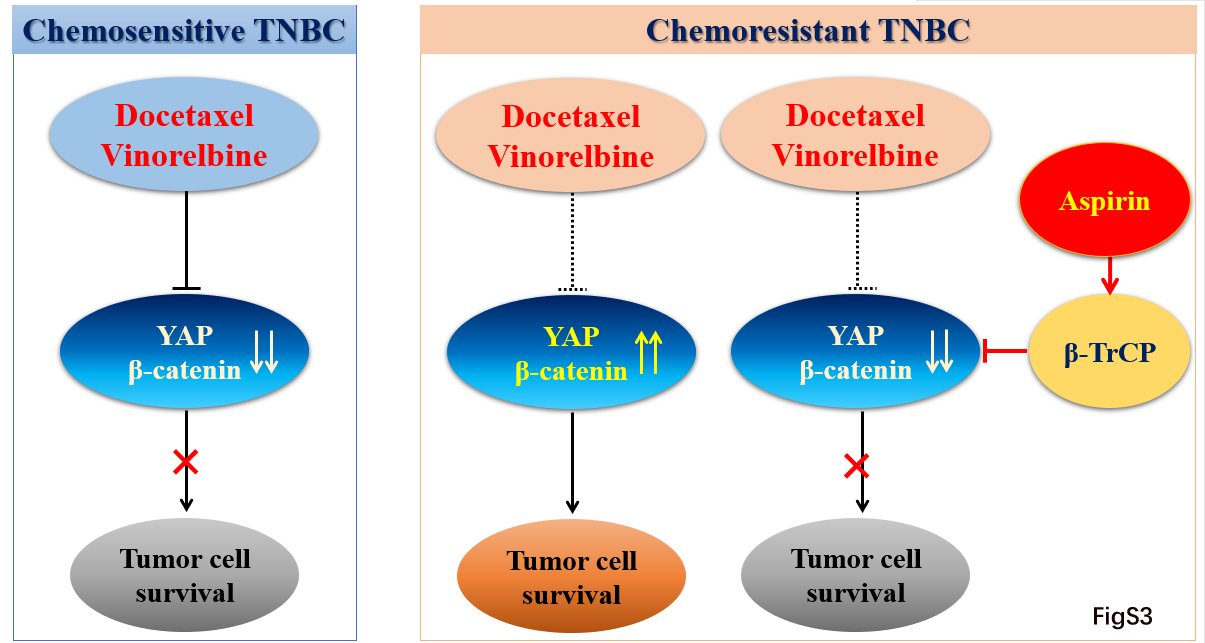

Supplement: Supplementary file 3 — FigureS3 [file 41419_2020_2719_MOESM3_ESM.tif]

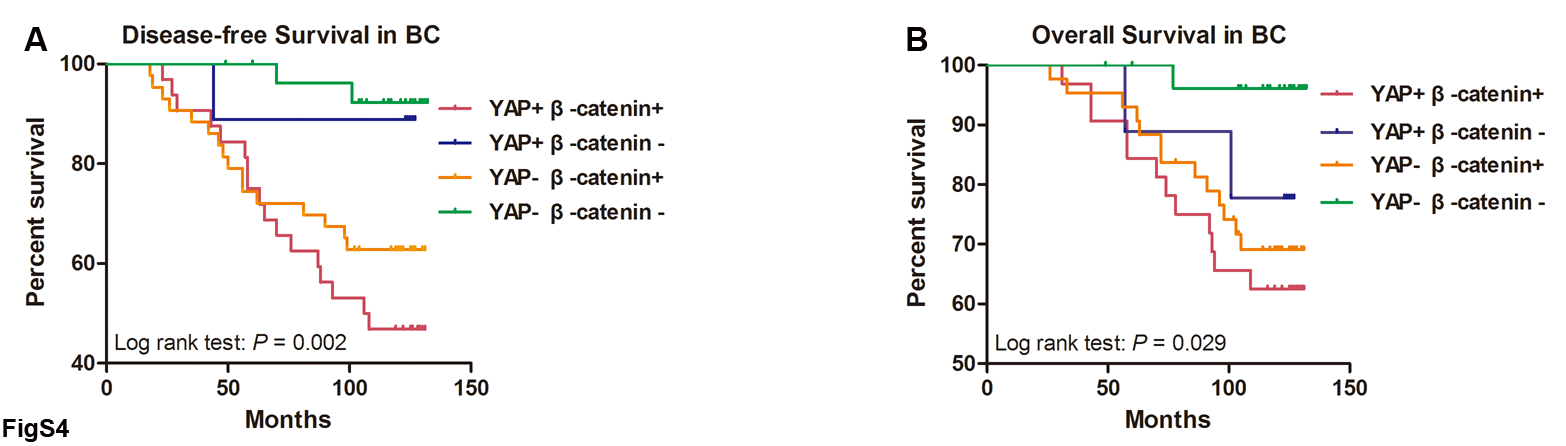

Supplement: Supplementary file 4 — FigureS4 [file 41419_2020_2719_MOESM4_ESM.tif]

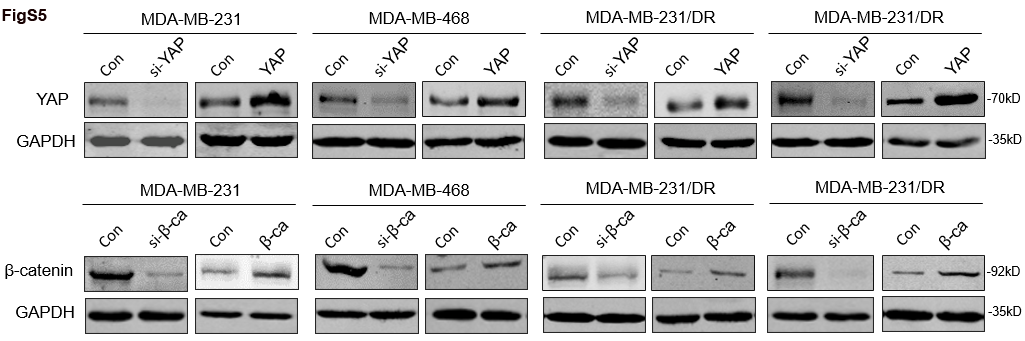

Supplement: Supplementary file 5 — FigureS5 [file 41419_2020_2719_MOESM5_ESM.tif]
