# Supplementary material for: Aspirin attenuates YAP and β-catenin expression by promoting β-TrCP to overcome docetaxel and vinorelbine resistance in triple-negative breast cancer
Source: Cell Death Dis. 2020 Jul 13;11(7):530. doi: 10.1038/s41419-020-2719-2 (PMC7359325; doi:10.1038/s41419-020-2719-2)
Supplement: Supplementary file 6 — Table.S1 [file 41419_2020_2719_MOESM6_ESM.doc]

**Table S1: Correlation of YAP or β-catenin expression with clinical pathologic characteristics in breast cancer specimens**

| Variable | YAP expression | | β-catenin expression | |
| --- | --- | --- | --- | --- |
|  | Correlation coefficient | *P* ^a^ | Correlation coefficient | *P* ^a^ |
| Age(years) | -0.086 | 0.367 | 0.007 | 0.971 |
| Tumor size | -0.224 | 0.018 | -0.006 | 0.953 |
| Lymph node metastasis | 0.198 | 0.036 | 0.207 | 0.029 |
| Histological grade | 0.057 | 0.548 | 0.199 | 0.036 |
| TNM stage | 0.169 | 0.076 | 0.194 | 0.041 |
| ER status | -0.024 | 0.802 | 0.059 | 0.535 |
| PR status | 0.087 | 0.363 | -0.062 | 0.518 |
| HER2 status | -0.033 | 0.733 | -0.061 | 0.521 |
| Molecular subtype | 0.026 | 0.789 | -0.066 | 0.490 |

^a^ The Spearman correlation test was used for statistical analyses. *P* values < 0.05 were considered statistically significant.
