# Supplementary material for: Aspirin attenuates YAP and β-catenin expression by promoting β-TrCP to overcome docetaxel and vinorelbine resistance in triple-negative breast cancer
Source: Cell Death Dis. 2020 Jul 13;11(7):530. doi: 10.1038/s41419-020-2719-2 (PMC7359325; doi:10.1038/s41419-020-2719-2)
Supplement: Supplementary file 7 — Table.S2 [file 41419_2020_2719_MOESM7_ESM.docx]

**Table S2: Univariate analyses of DFS and OS for patients with breast cancer**

| Variable | DFS | OS |
| --- | --- | --- |
|  | Log-rank  *P* | Log-rank  *P* |
| Age(years) (≤ 50 *vs* > 50) | 0.89 0.346 | 3.33 0.068 |
| Tumor size (≤ 2cm *vs* > 2cm) | <0.001 0.988 | 0.17 0.684 |
| Lymph node metastasis (negative *vs* positive) | 11.33 0.001 | 6.74 0.009 |
| Histological grade (I~II *vs* III) | 2.83 0.092 | 3.03 0.082 |
| TNM stage (I~II vs III~IV) | 18.39 <0.001 | 9.31 0.002 |
| ER status (negative *vs* positive) | 7.18 0.007 | 9.01 0.003 |
| PR status (negative *vs* positive) | 0.50 0.479 | 1.50 0.220 |
| HER2 status (negative *vs* positive) | 1.41 0.234 | 0.27 0.603 |
| YAP status (low *vs* high) | 3.29 0.070 | 2.56 0.110 |
| β-catenin status (low *vs* high) | 12.04 0.001 | 6.74 0.009 |
