# Supplementary material for: Aspirin attenuates YAP and β-catenin expression by promoting β-TrCP to overcome docetaxel and vinorelbine resistance in triple-negative breast cancer
Source: Cell Death Dis. 2020 Jul 13;11(7):530. doi: 10.1038/s41419-020-2719-2 (PMC7359325; doi:10.1038/s41419-020-2719-2)
Supplement: Supplementary file 8 — Table.S3 [file 41419_2020_2719_MOESM8_ESM.docx]

**Table S3: Multivariate analyses of DFS and OS for patients with breast cancer**

| Variable | DFS | OS |
| --- | --- | --- |
|  | HR 0.95CI  *P* | HR 0.95CI  *P* |
| Age(years) (≤ 50 *vs* > 50) | 2.18 1.04-4.56 0.039 | 3.67 1.48-9.13 0.005 |
| Tumor size (≤ 2cm *vs* > 2cm) | 0.77 0.36-1.64 0.497 | 1.04 0.44-2.47 0.952 |
| Lymph node metastasis (negative *vs* positive) | 1.13 0.23-5.47 0.884 | 1.71 0.33-8.82 0.522 |
| Histological grade (I~II *vs* III) | 1.58 0.76-3.29 0.218 | 1.37 0.60-3.13 0.450 |
| TNM stage (I~II vs III~IV) | 2.86 0.63-13.05 0.174 | 1.44 0.29-7.04 0.656 |
| ER status (negative *vs* positive) | 0.25 0.10-0.61 0.003 | 0.21 0.07-0.62 0.005 |
| PR status (negative *vs* positive) | 1.68 0.70-4.03 0.249 | 1.16 0.42-3.19 0.780 |
| HER2 status (negative *vs* positive) | 1.90 0.86-4.18 0.113 | 1.40 0.55-3.61 0.481 |
| YAP status (low *vs* high) | 1.55 0.73-3.28 0.257 | 2.19 0.93-5.15 0.073 |
| β-catenin status (low *vs* high) | 5.56 1.67-18.52 0.005 | 3.48 1.02-11.93 0.047 |
