# Supplementary material for: Aspirin attenuates YAP and β-catenin expression by promoting β-TrCP to overcome docetaxel and vinorelbine resistance in triple-negative breast cancer
Source: Cell Death Dis. 2020 Jul 13;11(7):530. doi: 10.1038/s41419-020-2719-2 (PMC7359325; doi:10.1038/s41419-020-2719-2)
Supplement: Supplementary file 9 — Supplementary figure legends [file 41419_2020_2719_MOESM9_ESM.docx]

**Fig. S1 The quantitative results of Fig. 3C.** The YAP and β-catenin protein expressions were quantified by Image J software. (A) MDA-MB-231 *vs* MDA-MB-231/DR. (B) MDA-MB-231 *vs* MDA-MB-231/VR. The data are presented as the mean ± SD of experiments performed in triplicate. Statistical significance was determined using Student’s *t* test analysis.

**Fig. S2 Inhibitory effects of aspirin on TNBC cells.** Proliferation assays at 24, 48, 72 and 96 hours for MDA-MB-231 and MDA-MB-468 cells cultured with different concentrations of aspirin (0, 2.5, 5, and 10 mM). (A) MDA-MB-231 cells (Asp 0 *vs* 2.5 mM, 5 mM, 10mM, 48 h group: *P=*0.037, *P=*0.010, *P<*0.001; 72 h group: *P=*0.023, *P=*0.002, *P<*0.001; 96 h group: *P=*0.032, *P<*0.001, *P<*0.001). (B) MDA-MB-468 cells (Asp 0 *vs* 2.5 mM, 5 mM, 10mM, 48 h group: *P=*0.265, *P=*0.012, *P=*0.004; 72 h group: *P=*0.030, *P<*0.001, *P<*0.001; 96 h group: *P=*0.010, *P<*0.001, *P<*0.001). Colony formation assays and qualitative analysis of MDA-MB-231 and MDA-MB-468 cells cultured with different concentrations of aspirin (0, 2.5, 5, and 10 mM) at day 10. (C) MDA-MB-231 cells (Asp 0 *vs* 2.5 mM, 5 mM, 10mM, *P=*0.007, *P<*0.001, *P<*0.001). (D) MDA-MB-468 cells (Asp 0 *vs* 2.5 mM, 5 mM, 10mM, *P=*0.011, *P<*0.001, *P<*0.001). Migration assays and qualitative analysis at 0, 24 and 48 hours for MDA-MB-231 and MDA-MB-468 cells cultured with different concentrations of aspirin (0, 2.5, 5, and 10 mM). (E) MDA-MB-231 cells (Asp 0 *vs* 2.5 mM, 5 mM, 10mM, 24 h group: *P=*0.012, *P<*0.001, *P<*0.001; 48 h group: *P=*0.003, *P<*0.001, *P<*0.001). Scale bar: 200 µM. (F) MDA-MB-468 cells (Asp 0 *vs* 2.5 mM, 5 mM, 10mM, 24 h group: *P=*0.158, *P<*0.001, *P<*0.001; 48 h group: *P<*0.001, *P<*0.001, *P<*0.001). Scale bar: 200 µM. The data are presented as the mean ± SD of experiments performed in triplicate. Statistical significance was determined using Student’s *t* test analysis.

**Fig. S3 A Schematic diagram of how aspirin overcomes chemotherapy resistance in TNBC.** Proposed working mode: in Chemosensitive TNBC, docetaxel or vinorelbine inhibits tumour cell survival, probably via the downregulating YAP and β-catenin expression. In Chemoresistant TNBC, YAP and β-catenin expression are increased and the inhibitive effect of docetaxel or vinorelbine on tumour cell survival is impaired, but aspirin can attenuate YAP and β-catenin expression by promoting β-TrCP to overcome docetaxel and vinorelbine resistance and kill the tumour cells.

**Fig. S4 Association between combined YAP and β-catenin expression and survival in breast cancer patients.** Kaplan–Meier analysis was carried out of DFS (A) and OS (B) according to combined YAP and β-catenin expression in breast cancer. Statistical significance was determined using log-rank analysis.

**Fig. S5 The expressions of YAP and β-catenin protein by western blot assay.** MDA-MB-231, MDA-MB-468, MDA-MB-231/DR and MDA-MB-231/VR cells were transfected by control siRNA, si-YAP, si-β-catenin, control plasmid, pCMV-Flag-YAP-5SA or pcDNA3.0-β-catenin, respectively. Then, the protein expressions were analysed by western blot. The data are presented from three independent experiments.
